# “Neural Network Responses to Traumatic Stress Predicting its Longterm Consequences”

**DOI:** 10.1192/j.eurpsy.2022.101

**Published:** 2022-09-01

**Authors:** B. Dirven, M. Negwer, J. Grandjean, J. Homberg, T. Kozicz, M. Henckens

**Affiliations:** 1 Radboud University Medical Center, Cognitive Neuroscience, Nijmegen, Netherlands; 2 Radboud University Medical Center, Human Genetics, Nijmegen, Netherlands; 3 Radboud University Medical Center, Radiology And Nuclear Medicine, Nijmegen, Netherlands; 4 Mayo Clinic, Laboratory Medicine And Pathology, Rochester, United States of America; 5 Mayo Clinic, Department Of Clinical Genomics, Rochester, United States of America; 6 Radboud University Medical Center, Medical Imaging, Nijmegen, Netherlands

**Keywords:** neural networks, animal model, resilience, PTSD

## Abstract

Adaptive responding to severe stress or trauma requires an optimized reconfiguration in the activity of large-scale neural networks. In vulnerable individuals, this response can go awry, inducing long-term consequences on mental health, such as posttraumatic stress disorder (PTSD). Improved understanding of the neurobiological mechanisms underlying this maladaptive neural response to trauma might benefit early intervention (i.e., secondary prevention) options in stress-related psychopathology. Yet, because of obvious ethical limitations these acute responses to trauma are inaccessible in humans. Therefore, we here used a mouse model for PTSD to investigate adaptive vs. maladaptive neural responding to trauma, the latter leading to long-term behavioral consequences mimicking symptoms observed in PTSD patients. By using transgenic mice, we were able to fluorescently label all activated neurons during trauma exposure, and relate these activation patterns to later PTSD-like symptomatology. We observed increased neuronal activity in sensory-processing and memory-related areas of mice vulnerable to the long-term consequences of trauma exposure, compared to resilient mice. Moreover, vulnerable mice displayed increased functional connectivity between the default mode network and lateral cortical network (a proxy for the central executive network in humans) during trauma processing relative to resilient mice. As such, these findings provide first insight in how a maladaptive neural response to trauma can result in later symptoms of psychopathology.

**Disclosure:**

No significant relationships.

